# Optimization of Turbidity and Organic Matter Removal from Palm Oil Mill Effluent by Batch Adsorption Using Gasification-Derived *Pinus patula* Biochar

**DOI:** 10.3390/molecules31193514

**Published:** 2026-10-02

**Authors:** Marlyn Y. Rivero-Mestra, Carolina Gallego-Ramírez, Ainhoa Rubio-Clemente

**Affiliations:** 1 Grupo de Investigación Energía Alternativa, Facultad de Ingeniería, Universidad de Antioquia UdeA, Calle 70 No. 52-21, Medellín 050010, Colombia; 2 Escuela Ambiental, Facultad de Ingeniería, Universidad de Antioquia UdeA, Calle 70 No. 52-21, Medellín 050010, Colombia

**Keywords:** biochar, wastewater, organic matter, circular bioeconomy, agro-industrial waste, oil palm

## Abstract

In the palm oil agroindustry, large volumes of POME (palm oil mill effluent) are generated during the oil extraction process, representing a significant source of water pollution by organic matter (OM) and turbidity. In this work, gasification-derived biochar from *Pinus patula* wood pellets was evaluated as an adsorbing material for POME treatment. For this purpose, a face-centred central composite design of experiments was used, and biochar dose and the pH of the solution were optimized through a second-order regression model. POME samples were taken from an agro-industrial company located in Colombia. The study identified a dose of biochar of 6 g/L and a pH of 6, as optimal operating conditions, achieving turbidity and OM in terms of chemical oxygen demand (COD) removal values of 58.27% and >50% at a treatment time of 30 min, respectively. The high specific surface area (367.33 m^2^/g) and pore volume (0.20 cm^3^/g) of the *Pinus patula* biochar used favoured turbidity and OM elimination mainly by physical mechanisms, such as pore filling and intraparticle diffusion, since the biochar surface remained near neutral at the optimal pH value found, minimizing electrostatic interactions. These results suggest that *Pinus patula* biochar has the potential to be used as an alternative technique to eliminate turbidity and decrease COD in POME. Additionally, it contributes to circular bioeconomy approaches through the valorisation of forestry solid waste in biochar production.

## 1. Introduction

Over the past five decades, palm oil has become the most widely consumed vegetable oil worldwide. Regardless of its economic benefits, its production generates large amounts of by-products, as only 10% of the processed biomass is converted into oil, while the remaining 90% becomes waste, including solid residues (e.g., empty fruit bunches, oil palm trunks, palm kernel shells, and pressed palm fibres) and a liquid effluent known as POME (palm oil mill effluent) [1]. Approximately, 2.5 t of POME are generated per t of oil extracted, and more than half of the water used in the process (5–7.5 t per t of oil) is released as POME, which consists of a thick, malodorous, and brown liquid [1,2,3]. Characterizations of POME have identified that it has an acidic pH (4–5), high organic matter (OM) content, biological and chemical oxygen demand (BOD and COD, respectively), total suspended solids (TSS), and turbidity [1]. Due to its complexity, the discharge of POME significantly alters the biological, chemical, and physical properties of the receiving water bodies [4].

In Colombia, the oil palm industry has grown significantly, positioning the country as the 4th largest palm oil producer behind Indonesia, Malaysia, and Thailand [5]. This situation, along with population growth, and insufficient sewage treatment systems, can pose significant pressure on its water resources [6,7,8]. For this reason, POME must be treated with an appropriate and efficient treatment before its discharged into the environment [3].

POME treatment technologies range from conventional methods, such as stabilization ponds, to more advanced multi-stage processes, integrating biological reactors [9]. Stabilization ponds are favoured for their cost-effectiveness [3]; however, they present limitations due to the high organic load, low pH (~4.5), and colloidal nature of suspended solids (SS) in POME, requiring large land areas, long hydraulic retention times, and generating additional environmental impacts, such as mosquito proliferation [9,10,11]. Natural coagulants, and anaerobic and aerobic biodegradation have also been evaluated [12,13]. For instance, Choong Lek et al. [10] achieved up to 86% turbidity, 56% COD, and 87% TSS removal using *Cicer arietinum* (chickpea) as a coagulant; nonetheless, COD removal efficiency decreased at higher doses and generated unsuitable sludge as fertilizer due to its low nutrient content [10,14]. In turn, biological systems, such as the sequential batch reactor (SBR), have achieved BOD removal efficiencies of 97–98%; nevertheless, despite achieving high OM removal, these systems require rigorous operating conditions with high operational and maintenance costs associated [15].

Among the alternative techniques that have been evaluated to treat POME, adsorption is highlighted due to its viability, high efficiency, easy operation, cost-effectiveness, and simple design requirements [12,13,16]. Specifically, adsorption has been used to remove SS, high-density metals, and residual oil [15]. The efficiency of adsorption in the treatment of POME is highly dependent on contact time, adsorbent type and dose, and the properties of the pollutants present in POME [17]. Various adsorbents, including clay, zeolite, nanomaterials, activated carbon, and biochar, have been studied [12]. Among these, carbon-based materials, like biochar, are especially valued for their low cost, ease of processing, stability, and tuneable porosity, making it a preferred choice in adsorption applications [18]. Additionally, the use of biochar contributes to circular bioeconomy models by transforming waste into raw material for new production processes, thus promoting economic and environmental sustainability [19].

Biochar is a solid, carbon-rich, porous material produced through the thermochemical conversion of biomass, such as wood, forestry and agricultural by-products, sewage sludge, or manure, using technologies that includes pyrolysis, gasification, hydrothermal carbonization, and torrefaction, among others [20,21]. Biochar has become a promising material for wastewater (WW) treatment due to its exceptional adsorbent properties, high surface area, porosity, and oxygen-containing functional groups (e.g., hydroxyl, carbonyl, and carboxyl) [22]. These properties largely depend on the feedstock source and the thermochemical production method [23]. In fact, biochar produced via gasification exhibits larger pore volume and surface area than pyrolysis-derived biochar, due to the higher temperatures involved in the process and the release of volatile organic and aliphatic compounds [24], while lignocellulosic feedstocks, such as wood, yield biochar with a well-defined porous structure, owing to the volatile nature of cellulose content [25].

*Pinus patula* is a tree species covering approximately 38,500 ha in Colombia, with a harvest cycle of approximately 13 years and an average yield of 20 m^3^/ha·year. These characteristics give this species potential for energy generation [26] and the reuse of its residual biomass, such as biochar. Accordingly, it is relevant to evaluate biochar derived from *Pinus patula* as an alternative treatment for contaminated water, thereby preventing the release of toxic substances into the environment.

Different studies have explored the utilization of biochar from forestry and agricultural residues for the treatment of POME. In one of these studies, empty oil palm fruit bunches were transformed into high specific surface area biochar, achieving a removal efficiency of 55% for COD [27]. Another research used biochar produced from the pyrolysis of orange and banana peels at temperatures between 400 °C and 500 °C. This biochar proved to be highly efficient as an adsorbent, reducing COD values by almost 57% [28].

Despite the high efficiencies reported in the literature, most of the studies based on the evaluation of biochar to treat WW are focused on the use of pyrolysis-derived biochar [29] and, from the authors’ knowledge, research evaluating the gasification-derived biochar to treat POME are scarce. Considering that gasification yields a biochar with a larger surface area and has shown to be effective in the removal of inorganic and organic pollutants from WW, it is crucial to evaluate the efficiency of biochar produced through the referred thermochemical process on the treatment of POME [30]. Furthermore, most studies reported focused on optimizing POME treatment system rely on one-factor-at-a-time technique. Nonetheless, this technique does not allow for the evaluation of interactions between different operational factors that can influence the efficiency of the process under study and, therefore, the presence of synergies and antagonisms between the levels of the different parameters evaluated. Furthermore, one-factor-at-a-time approach requires a significant amount of experimentation, with the subsequent associated costs and time, and it cannot optimize several factors simultaneously. In this regard, the use of response surface methodology (RSM) through experimental designs is recommended.

In this context, the present work studies the utilization of biochar produced from *Pinus patula* gasification for treating WW generated by an oil palm agroindustry in Colombia. Specifically, it aims to assess its efficiency as an alternative technology for turbidity and OM removal from POME. The influence of operational parameters, including pH and biochar dose, is analyzed employing the RSM, particularly a central-composite design of experiments (CCD). This research aims to provide an adapted solution that promotes environmental protection, economic sustainability, and an improved quality of life.

## 2. Results and Discussion

### 2.1. Palm Oil Mill Effluent (POME) Characterization

The POME used in this study was derived from a conventional treatment process within the agro-industry, consisting of a series of ponds, so that the liquid effluent generated from the oil palm extrusion process enters, first, to an equalization reactor, then goes through an anaerobic pond and, finally, through a facultative pond, before being discharged into the environment. In Table 1, POME physicochemical characteristics are compiled. As shown in the table, POME exhibited a turbidity of 296 ± 8 NTU, which is attributed to its SS content. While POME typically has an acidic pH, anaerobic treatment increases the pH to a range between 7.5 and 7.8. Additionally, the turbidity of anaerobically treated POME falls within the range of 220–600 NTU, which is comparable to the values obtained in the present study [2,31,32,33]. Furthermore, high OM levels (15,400 ± 1758.63 mg O_2_/L) were registered.

### 2.2. Pinus patula Biochar Characterization

*Pinus patula* biochar was characterized through proximate and ultimate analyses, specific surface area, pore volume, scanning electron microscopy (SEM), Fourier transform infrared spectroscopy (FTIR) and Raman spectroscopy, and thermo-gravimetric analysis (TGA). Table 2 presents the results for the ultimate and proximate analyses, where carbon (C), nitrogen (N), oxygen (O), hydrogen (H), volatile matter, ash, and moisture contents were listed, as well as the specific surface area and pore volume findings obtained for *Pinus patula* biochar and wood pellets. As detailed in Gutiérrez et al. [26], the porous structure of *Pinus patula* biochar was enhanced due to the release of volatile matter with regard to the raw biomass. This contributed to the formation of pores and the increase in the surface area, which can also be corroborated by the SEM image results. In Gutiérrez et al. [26], SEM analysis shows the formation of amorphous carbonaceous structures after the gasification of *Pinus patula* wood pellets. In the referred study, the formation of pores, which enhances the adsorption capacity of *Pinus patula* biochar, can also be identified.

According to FTIR characterization results reported for the biochar [26], the intensity of oxygen-containing functional groups (–OH, –COOH, and –CO) decreased after the gasification process; this change is associated with the release of volatile matter and moisture during the thermochemical conversion of the raw biomass. Additionally, following gasification, the peak at 1520 cm^−1^ corresponding to C=C bond stretching disappeared, indicating the development of an aromatic structure in the biochar [34].

To confirm the thermal stability of *Pinus patula* biochar, a TGA was conducted as presented in [26]. It was identified that the raw *Pinus patula* wood pellets showed a marked mass loss at 250 and 400 °C, associated with the decomposition of hemicellulose and cellulose. Likewise, *Pinus patula* biochar presented a lower mass loss within that temperature range, since cellulose and hemicellulose were already decomposed during the gasification process. In contrast to the raw biomass, the biochar did not show a defined base temperature in the derivative thermogravimetric curve (DTG) [26], indicating that *Pinus patula* biochar presents higher thermal stability and lower reactivity than raw biomass. These results allowed us to confirm that *Pinus patula* biochar is a thermally stable and carbonized material.

Finally, with regard to Raman spectroscopy, the adsorbing material exhibits an ID/IG index higher than 0.80, which indicates the high degree of defects, and the subsequent enhancement of the adsorption capacity of the biochar derived from *Pinus patula* wood pellets used.

### 2.3. POME Treatment Using Pinus patula Biochar

Based on the type of experimental design, which consisted of a CCD with two operational parameters and three levels per parameter, a 3^2^ design was conducted with three central points. The resulting matrix is described in Table 3. As observed, it consisted of 11 treatments. Once all 11 treatments were randomized to avoid data bias [34], an ANOVA (analysis of variance) was performed to evaluate the effects of the operating parameters selected and to represent the removal of turbidity from POME using *Pinus patula* biochar by a second-order regression model.

**Figure 1:**
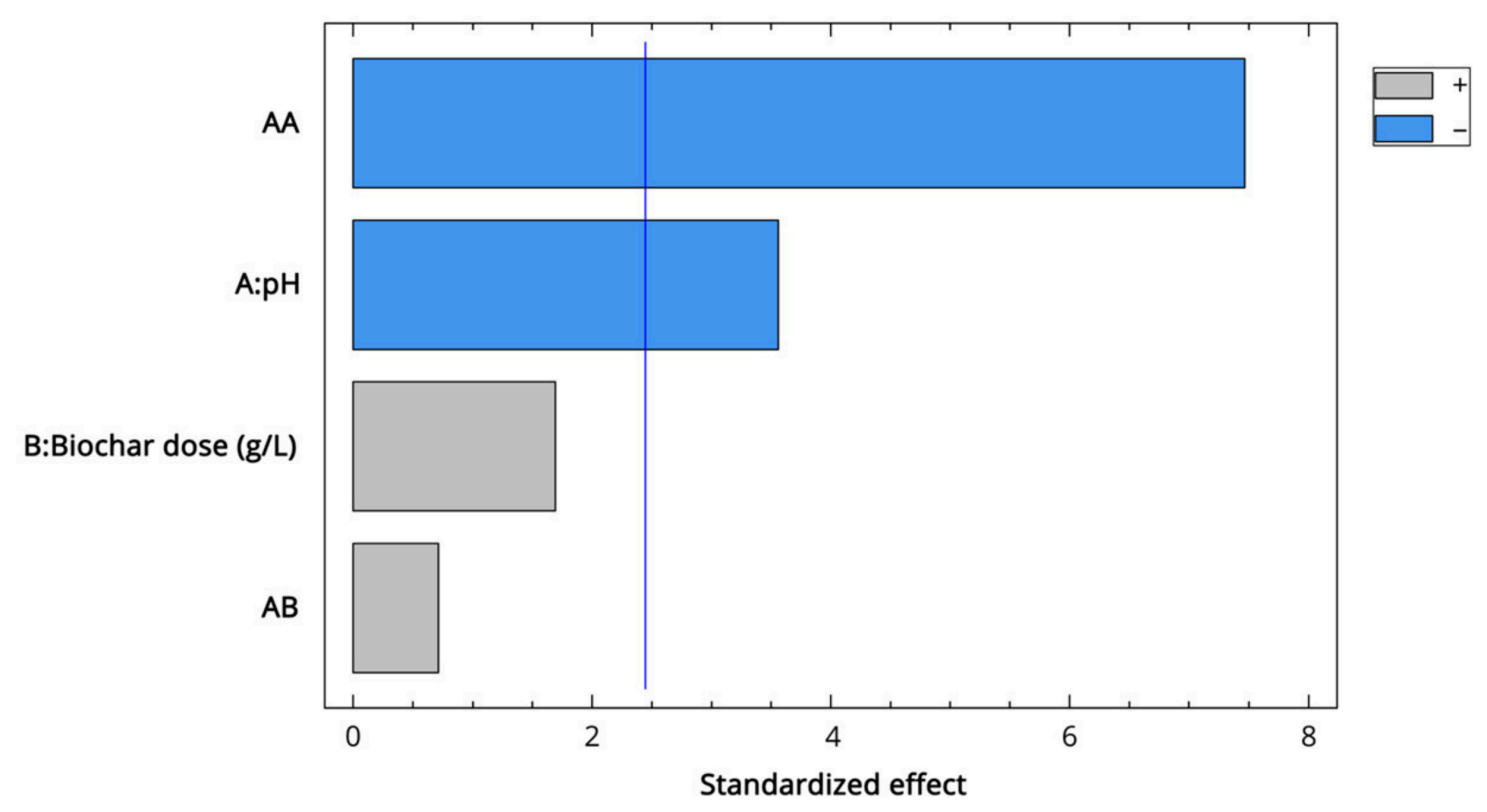
Pareto chart for the elimination of turbidity from POME using *Pinus patula* biochar. Operating conditions: pH = 9, 6, and 3; biochar dose = 2, 4, and 6 g/L; contact time = 30 min; biochar particle size = 375 µm.

By carrying out the ANOVA (Table 4), it was found that pH and its quadratic effect were statistically significant, since the associated *p*-values were <0.05 (significance level of 5%). Neither the main effect of biochar dose nor the interaction effect between pH and biochar dose reached statistical significance. Consequently, the impact of each factor on the response variable remains consistent across the levels of the other factor [35]. The ANOVA yielded a *p*-value below 0.05, which supports the conclusion that the medians are statistically different and that the observed differences are not due to random variation [36]. In the Pareto chart, which is presented in Figure 1, the quadratic effect of pH emerged as the most significant factor affecting the adsorption system for turbidity removal from POME. The main effect of pH was identified as the second-most significant parameter in the removal of turbidity using *Pinus patula* biochar. Additionally, the interaction between biochar dose and solution pH was the least influential parameter in the adsorption system.

**Figure 2:**
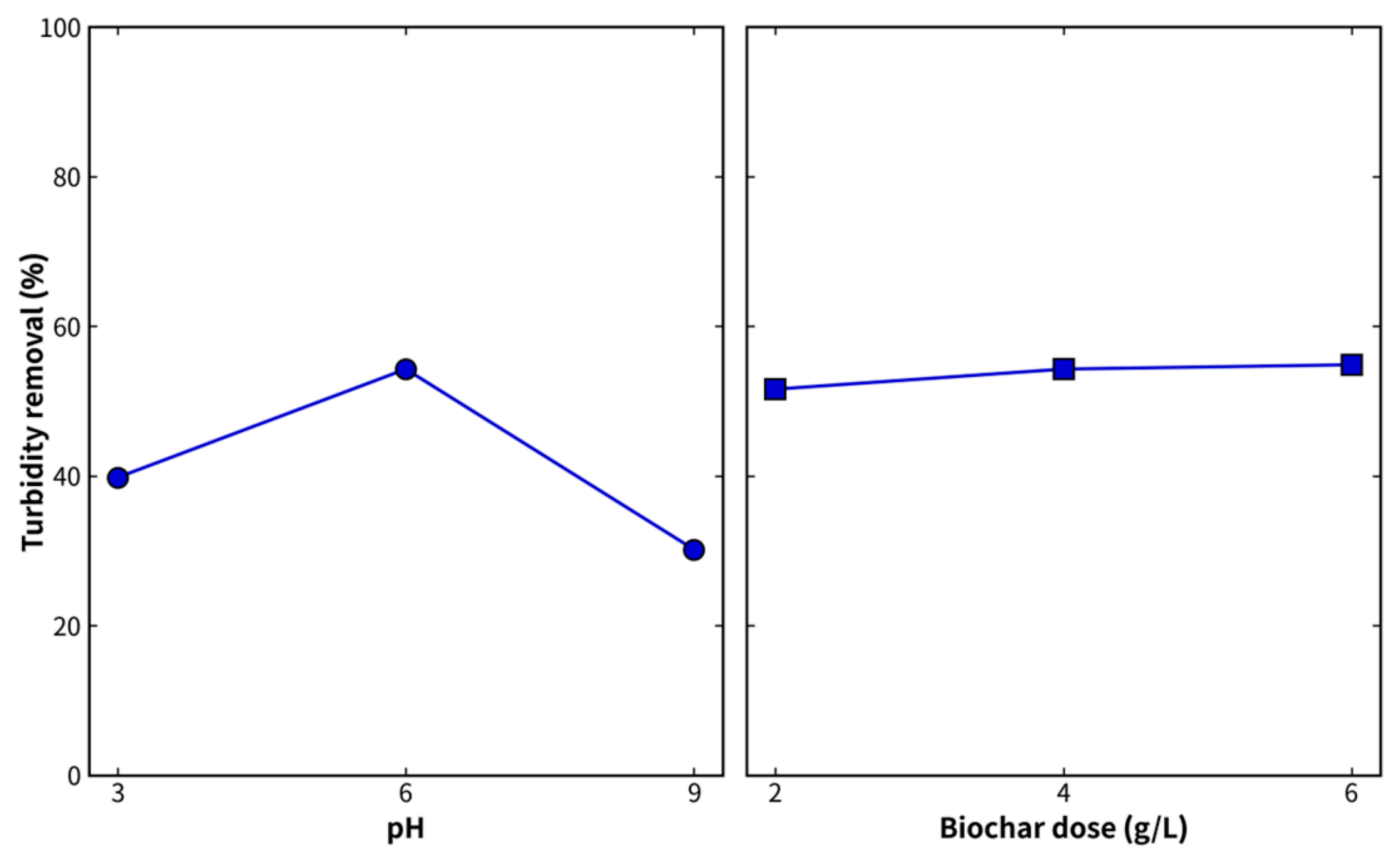
Main effects chart for the elimination of turbidity from POME using *Pinus patula* biochar. Operating conditions: pH = 9, 6, and 3; biochar dose = 2, 4, and 6 g/L; contact time = 30 min; biochar particle size = 375 µm.

In Figure 2, the main effects of the two evaluated parameters, solution pH and biochar dose, can be observed. Regarding the pH, it is shown that when this factor is located at the centred point (i.e., 6), the elimination efficiency is enhanced. Considering the point of zero charge (PZC) of the biochar under study (PZC = 6) and that the turbidity removal is enhanced at a solution pH of 6, it is suggested that biochar presents more efficiency removing suspended particles when its charge is neutral. This can also infer that the response surface of the adsorption system describing the removal of turbidity from POME by *Pinus patula* biochar has a curvature and that the quadratic effect of pH has statistical significance, as informed by the Pareto chart (Figure 1) and ANOVA (Table 4). Regarding *Pinus patula* biochar dose, the results obtained from Figure 2 indicates that an increase in the dose of the adsorbent is proportional to a slight increase in the turbidity elimination efficiency.

#### 2.3.1. Effect of the pH of the Solution

Concerning the operational parameters evaluated, solution pH was found to be the most significant factor affecting the elimination of turbidity from POME using *Pinus patula* biochar. The performance of an adsorption process is highly dependent on solution pH, since pH can influence biochar surface charge and the ionic forms of the suspended particles [37]. pH can cause deprotonation or protonation of the functional groups on the surface of the biochar, thus influencing its ability to adsorb particles on its surface [38]. To evaluate the influence of pH in the present study, pH values of 3, 6, and 9 were studied. By conducting the experimental design, it was found that the optimal value of pH that maximized the removal of turbidity from POME was 6. At the highest and lowest pH values assessed (9 and 3, respectively), the turbidity removal efficiency showed a decrease. Considering the PZC value of *Pinus patula* biochar (PZC = 6), at a solution pH of 6 the biochar is at its isoelectric point, therefore the positive charges in the biochar surface balance the negative charges, resulting in a biochar with a neutral surface [39,40].

SS in water usually present a negative charge, which promotes their attraction to adsorbents with positively charged surfaces [41]. However, since the highest turbidity removal efficiency was attained when the surface of *Pinus patula* biochar was neutral, it can be inferred that electrostatic attraction was not the primary mechanism in eliminating turbidity-causing particles in POME [42]. In contrast, the removal of turbidity from POME may be attributed to physical adsorption forces, such as pore filling, intraparticle diffusion of SS into the biochar, and co-precipitation [43]. Moreover, the large surface area (367.33 m^2^/g) and pore volume (0.20 cm^3^/g) of *Pinus patula* biochar [44] enhance its capacity to remove SS from water by physical mechanisms, as its porous structure enables the retention and subsequent elimination of SS from the aqueous solution [26,45].

A similar behaviour was observed in the study conducted by James et al. [45] on POME treatment using a biochar-based nanocomposite activated with montmorillonite clay and titanium dioxide (TiO_2_) (Table 5). As the pH of POME approached neutrality, interactions between the nanocomposite and the contaminants intensified due to the absence of repulsive forces, consistent with the results observed in the present study. In contrast, Razali and Kamarulzaman [30] reported lower COD removal using biochar derived from oil palm trunk under basic conditions (Table 5), highlighting the importance of pH optimization for improving pollutant removal, minimizing material use, and reducing treatment costs and time.

#### 2.3.2. Effect of *Pinus patula* Biochar Dose

In the adsorption process, the adsorbent dose directly influences the capacity to remove pollutants from water. Furthermore, the adsorbent dose is a parameter that requires optimization, as it can significantly impact the overall cost of process implementation [50,51]. When the adsorbent dose is below its optimal value, there is insufficient surface area and an inadequate number of active sites on the biochar to effectively remove SS from WW. On the other hand, when the biochar dose exceeds the optimum, biochar particle collisions may induce the desorption of previously adsorbed SS from the biochar surface. Additionally, higher doses increase the diffusion path length, hindering particles from reaching the active adsorption sites and thereby extending the time required to achieve maximum removal efficiencies [44].

In the present study, the effect of *Pinus patula* biochar dose was evaluated over a range from 2 to 6 g/L. As shown in Figure 2, turbidity removal efficiency from POME increases with higher biochar doses, with an optimal dose determined at 6 g/L within the evaluated experimental domain. At this dose, the rise in biochar active sites and surface area significantly enhances turbidity removal from POME [52,53]. A similar behaviour was reported by Mubarak et al. [54] during POME treatment. The referred authors found that increasing the dose of the activate carbon derived from the oil palm kernel shell (OPKS-AC) led to significant enhancements in the elimination of BOD, COD, SS, and colour. Specifically, at lower doses (50 g/L), removal efficiency was limited due to insufficient active sites relative to the adsorbate concentration, whereas at higher doses (250 g/L), removal efficiencies reached 91%, 87%, 74%, and 97%, respectively, indicating a clear enhancement in performance until equilibrium was attained [54]. Likewise, Mekpan et al. [55] reported that employing fungal pellets as bioadsorbents in POME treatment resulted in an increased elimination efficiency with higher adsorbent doses, which was attributed to the increased surface area and the availability of additional binding sites [55]. In turn, Hayawin et al. [56] found the pollutant adsorption potential of OPKS-AC with an increased adsorbent concentration during POME treatment.

**Figure 3:**
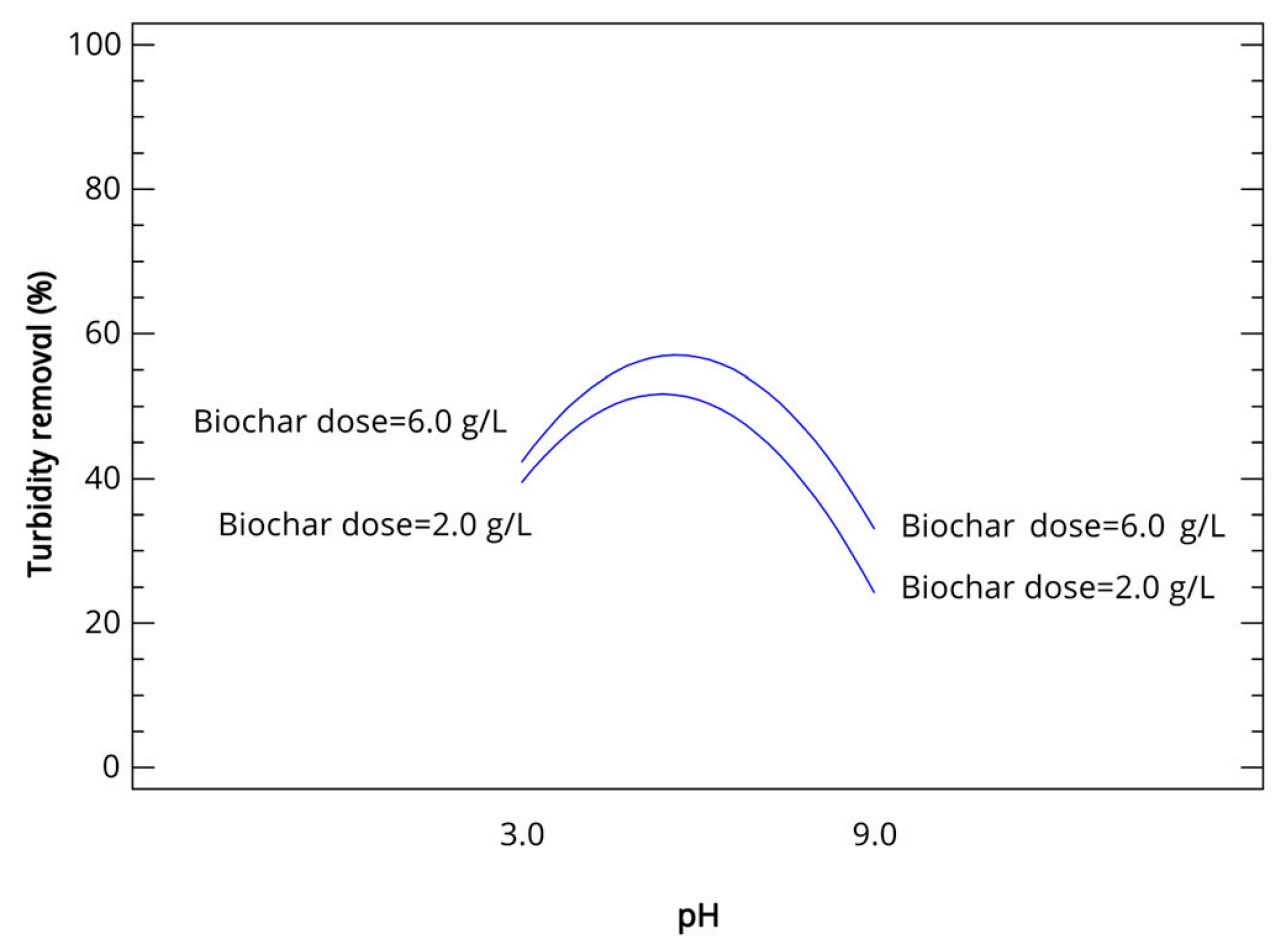
Interaction plot of pH and biochar dose for the elimination of turbidity from POME using *Pinus patula* biochar. Operating conditions: pH = 9, 6, and 3; biochar dose = 2, 4, and 6 g/L; contact time = 30 min; biochar particle size = 375 µm.

As presented in Table 4 (ANOVA) and the Pareto chart (Figure 1), the interaction between pH and biochar dose was not statistically significant. Figure 3 presents the interaction plot between these two operational factors, confirming that no direct interaction exists between them [35]. Regardless of biochar dose, the maximum removal efficiency for the process is obtained at a solution pH of 6. Furthermore, it can also be observed that the turbidity removal efficiency is enhanced when *Pinus patula* biochar dose is 6 g/L.

### 2.4. Optimization of the Process and Validation of the Regression Model

**Figure 4:**
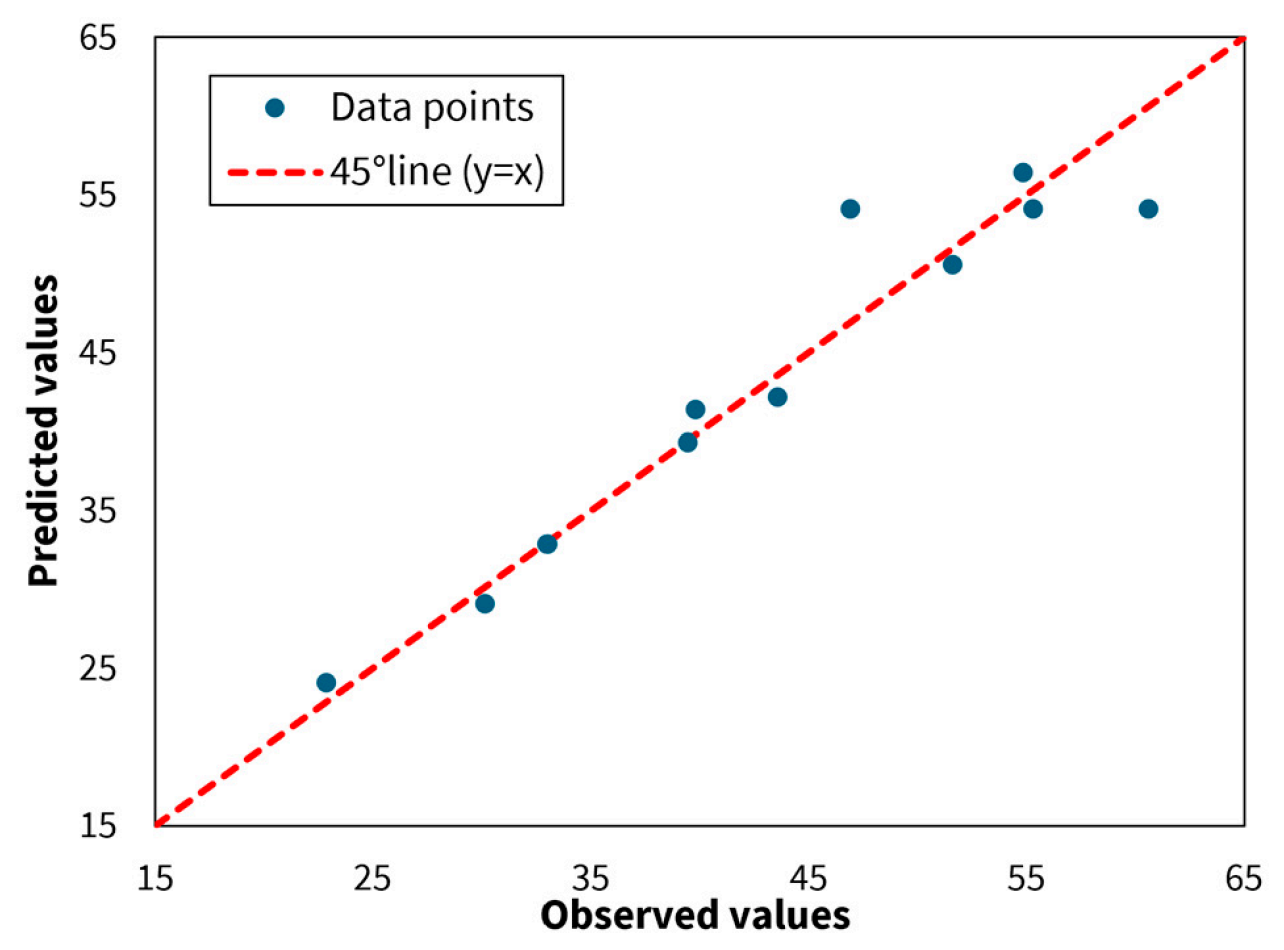
Predicted vs. observed values for turbidity removal from POME using *Pinus patula* biochar. Operating conditions: pH = 9, 6, and 3; biochar dose = 2, 4, and 6 g/L; contact time = 30 min; biochar particle size = 375 µm.

**Figure 5:**
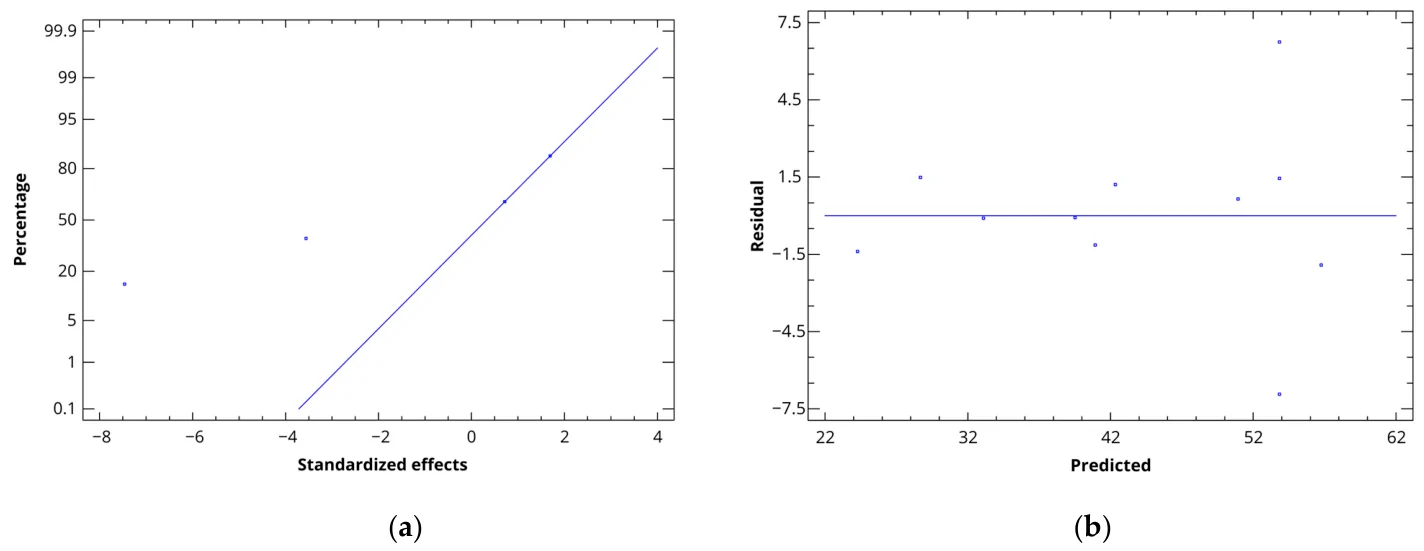
(**a**) Normal probability plot and (**b**) Independence of the residuals plot for the elimination of turbidity from POME using *Pinus patula* biochar. Operating conditions: pH = 9, 6, and 3; biochar dose = 2, 4, and 6 g/L; contact time = 30 min; biochar particle size = 375 µm.

To describe the removal of turbidity from POME by using *Pinus patula* biochar, a quadratic regression model was developed. As observed in Table 6, the model *p*-value indicates that the quadratic model built is statistically significant and provides a good fit for representing turbidity removal by *Pinus patula* biochar within the selected experimental domain. R^2^ and R^2^_adj_ values of 93% and 87%, respectively, demonstrate that the regression model accounts for a substantial proportion of the data variability. Additionally, as presented in the predicted vs. observed values (Figure 4), there is a good agreement between the observed (experimental) values and the turbidity removal values predicted by the model, since data points are closely distributed along the reference line (45° line). Nonetheless, the hypotheses of normality, independence of the residuals and homoscedasticity were validated to corroborate the regression model adequacy. For normality hypothesis clarification, a normal probability plot (Figure 5a) was constructed. As illustrated in the figure, most points followed the reference line, except for the first two ones. In addition, Shapiro–Wilk test had an associated *p*-value > 0.05, supporting normality assumption. To assess the independence of residuals, a residual plot (Figure 5b) and Durbin–Watson test were employed. The plot revealed that data do not follow a systematic pattern, and Durbin–Watson test yielded a *p*-value above 0.05, confirming the independence of residuals. In turn, Breusch–Pagan test for homoscedasticity returned a *p*-value above 0.05, verifying that the homoscedasticity assumption was met by the second-order regression model constructed. Finally, the *p*-value associated with the regression model was 0.0017, confirming the significance of the model constructed for explaining the adsorption system under the experimental domain.

Therefore, by satisfying the hypotheses of homoscedasticity, independence of residuals, and normality, the reliability of the second-order regression model obtained from the CCD experimental design was confirmed [38]. The resulting second-order regression model is presented in Equation (1), where *T* stands for the elimination of turbidity, and *x*_1_ and *x*_2_ refers to the solution pH and biochar dose, respectively.

(1) $$T=-9.89333+22.35x_{1}-0.0466667x_{2}+0.250417x_{1}x_{2}-2.11611{x_{1}}^{2}$$

**Figure 6:**
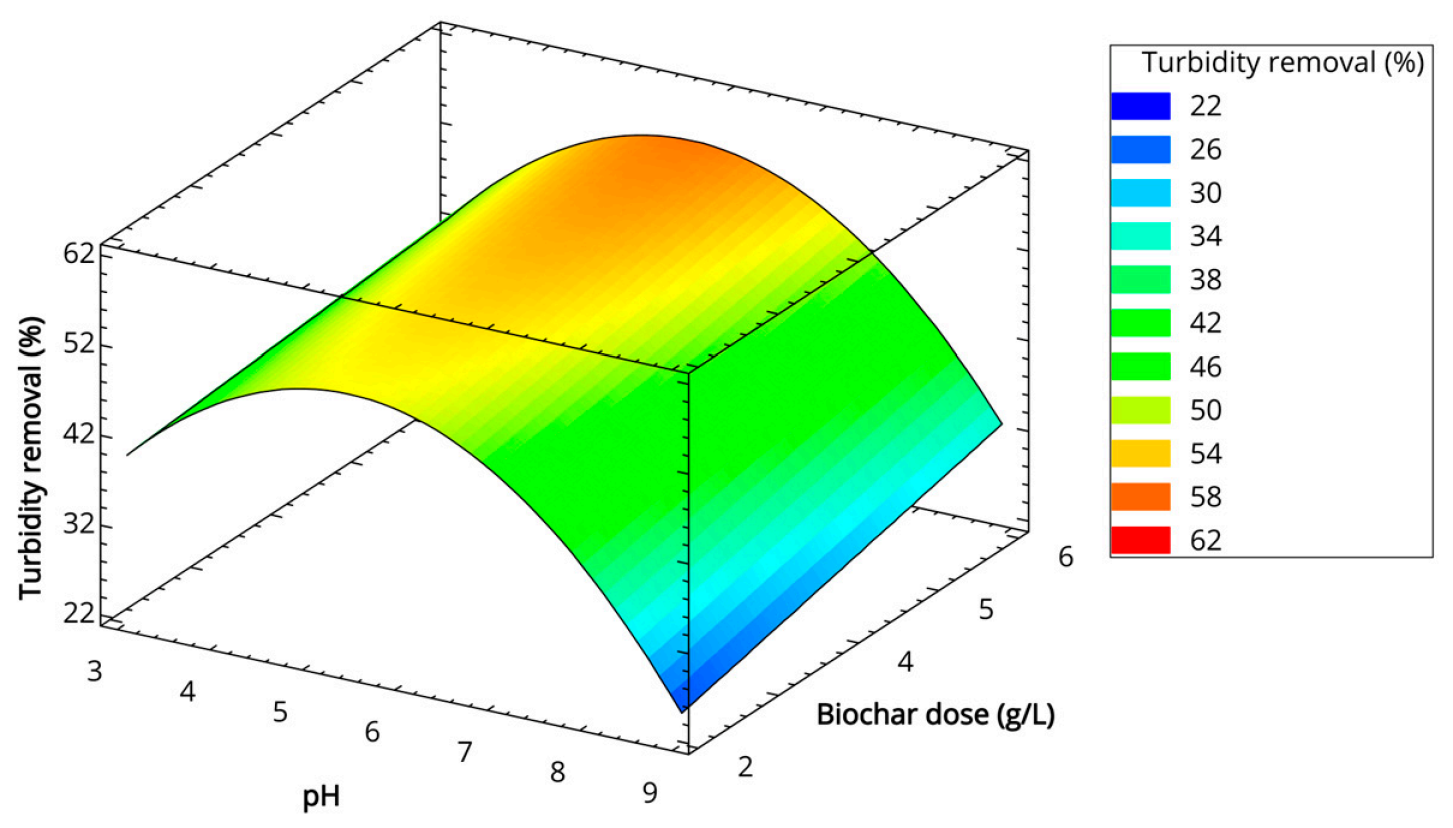
Response surface for the elimination of turbidity from POME using *Pinus patula* biochar. Operating conditions: pH = 9, 6, and 3; biochar dose = 2, 4, and 6 g/L; contact time = 30 min; biochar particle size = 375 µm.

To discern the optimal operational parameters for the removal of turbidity from POME using *Pinus patula* biochar, a response surface was generated from the quadratic regression model. As depicted in Figure 6, maximum removal efficiency values were achieved at a solution pH of 6 and a biochar dose of 6 g/L. When the pH was maintained at 6, the influence of the biochar dose was less pronounced, with removal efficiencies ranging between 54% and 62% regardless of the biochar dose. However, when the pH deviated from the optimum, the effect of the dose of biochar became more significant, with lower doses leading to a marked decrease in the process efficiency [50]. The optimal conditions were further confirmed using the desirability function.

**Figure 7:**
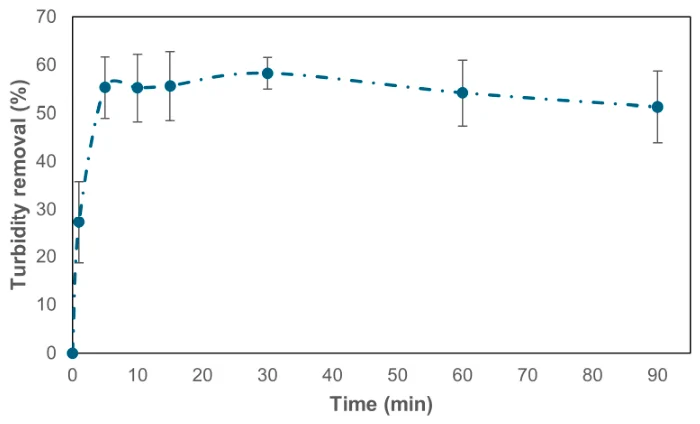
Turbidity elimination from POME using *Pinus patula* biochar under optimal conditions. Operating conditions: pH = 6; biochar dose = 6 g/L; biochar particle size = 375 µm.

Additional treatments (n = 3) were conducted to evaluate turbidity removal from POME under optimal conditions (i.e., a solution pH of 6 and a biochar dose of 6 g/L). Treatments were performed with a contact time of 90 min, and aliquots were withdrawn at 0, 1, 5, 10, 15, 30, 60, and 90 min. As illustrated in Figure 7, the removal efficiency reached 55.34% at 5 min, with the maximum efficiency of 58.27% achieved at 30 min. Initially, the high concentration of SS in POME resulted in an elevated turbidity and provided a strong driving force for rapid adsorption. Following this initial phase, the adsorption rate decreased due to slower diffusion of SS into the surface and pores of the *Pinus patula* biochar and the saturation of its active sites [55]. As observed in Figure 7, after 30 min, the elimination efficiency began to decline, which can be attributed to the inherent instability of physical mechanisms of adsorption, such as pore filling. This instability may facilitate the desorption of adsorbed suspended particles, thereby increasing POME turbidity and reducing the overall removal efficiency [57]. In this regard, in order to confirm these results, further studies need to be conducted to discern whether the reduction in elimination efficiency of the process over time is due to the desorption of previously adsorbed particles in biochar surface.

Table 7 presents various studies evaluating the removal of turbidity from POME using different processes. Electrocoagulation, flocculation–coagulation, and ultrafiltration have demonstrated high turbidity removal efficiencies. Nevertheless, each method presents certain limitations. For instance, electrocoagulation produces acidic by-products that require pH neutralization prior to discharge to prevent adverse effects on aquatic organisms [1]. In turn, although peroxi-coagulation can achieve high removal efficiencies in heavily polluted water, it is associated with the generation of toxic by-products and the production of large volumes of sludge that must be properly disposed [2]. Flocculation–coagulation using natural flocculants is challenged by the rapid degradation of these agents and the subsequent sludge production [58]. Finally, despite its high efficiency in turbidity removal, ultrafiltration is limited by membrane fouling, which reduces efficiency over time and leads to increased costs during operation due to the need for regular membrane cleaning [59].

Even though the treatment of POME with *Pinus patula* biochar results in lower turbidity removal efficiencies compared to the aforementioned methods, biochar remains a promising material for WW treatment. To discern the performance of the process for OM elimination, at optimal biochar dose (6 g/L) and solution pH (6), the abatement of COD was studied after 90 min of contact time. It was found that a COD decreased from 15,400 to 7546 mgO_2_/L, corresponding to a removal higher than 50% of the organic compounds contained in the POME using *Pinus patula* biochar. In addition to the removal efficiency, the adsorption capacity (q_t_) of *Pinus patula* biochar was calculated for COD removal at optimal conditions (pH = 6 and biochar dose = 6 g/L). Under these conditions, an adsorption capacity (q_t_) of 1309 mg/g was obtained. The value of q_t_ found reflects the high adsorption capacity of the biochar used, which could be related to the high ID/IG index derived from Raman spectroscopy, and the large surface area and number of pores, along with the other physico-chemical characteristics of the adsorbing material used in the current study.

Despite this comparatively lower removal efficiency, *Pinus patula* biochar offers practical advantages that support its use as a sustainable alternative for POME treatment. In this regard, since biochar is produced from residual biomass, it has low production costs, which translates into reduced overall operational expenses [61]. For instance, the estimated production cost of biochar ranges from 100 to 600 USD per t, depending on the thermochemical method used for its production and its associated energy consumption, which is considerably lower than the cost of activated carbon (600 to 1000 USD per t) [62,63]. Furthermore, high removal values of turbidity and OM were obtained at a relatively low biochar dose (6 g/L), which reduces the amount of solid residue generated during the treatment. Nevertheless, the spent biochar still requires appropriate management, such as its use for energy recovery or as a soil amendment after evaluating the toxicity potential of the spent biochar [64,65]. Moreover, the *Pinus patula* biochar process operates at a near-neutral pH, thereby eliminating the need for effluent neutralization after treatment. Considering that most studies on biochar efficiency in WW treatment have been conducted using synthetic water, there is a clear need to evaluate its performance in real WW systems, as carried out in the current research. This approach would broaden the applicability of biochar and enhance the understanding of pollutant removal mechanisms in real WW matrices.

Nevertheless, a limitation of the present study is that the regeneration and reusability of *Pinus patula* biochar was not evaluated. Therefore, future studies should assess the feasibility of regenerating *Pinus patula* biochar through washing, thermal, and chemical treatments, as well as its performance over multiple adsorption–desorption cycles, to further strengthen the economic feasibility and sustainability of the adsorption treatment approach for POME.

In addition, future research should focus on the evaluation of adsorption kinetics for turbidity and COD removal from POME using *Pinus patula* biochar, to complement the optimization results obtained in this study. This analysis is essential for scaling up the treatment and estimating the contact time required for industrial applications, as it provides information about the adsorption rate and the potential rate-limiting steps involved in the process.

Addressing these aspects would further strengthen the feasibility of POME treatment with *Pinus patula* biochar.

## 3. Materials and Methods

### 3.1. POME and Reagents

Sodium hydroxide (NaOH, >99% purity) and hydrochloric acid (HCl, 37% purity) were utilized for adjusting the pH of the sample and were purchased from Merck KGaA (Darmstad, Germany). All reagents were used during the experiment without prior purification.

On the other hand, POME was sampled from an oil palm production company located in Colombia after being treated with a pond system, consisting of an equalization pond, an anaerobic pond and a facultative pond.

### 3.2. Biochar Production and Characterization

Biochar was produced through the gasification of wood pellets from *Pinus patula* in a top-lit updraft (TLUD) equipment that was fed with air as the gasification agent. The wood pellets, measuring 8 mm and 15–10 mm in diameter and length, respectively, were sourced from a local sawmill located in Medellín (Colombia). Detailed information on the gasification process is available in Rubio–Clemente et al. [34] and Gutiérrez et al. [26]. After biochar production, it was cooled down, crushed and sieved to obtain a particle size of 375 µm and stored until its used in zipper bags. Additionally, it was physico-chemically characterized by ultimate and proximate analyses, pore volume, specific surface area, SEM, FTIR, TGA, and Raman spectroscopy. In [26,34], further information on the specific methods and equipment used to discern the physico-chemical properties of the adsorbing material used are detailed.

### 3.3. POME Sampling, Characterization, and Treatment

A volume of 40 L of POME was collected from the control point (discharge) of the palm oil industry. The sample was transferred to a polyethylene container and kept at 4 °C to preserve POME physico-chemical characteristics. POME was characterized measuring the initial value of turbidity, pH, conductivity, temperature, and COD. Batch experiments were conducted to assess the efficiency of *Pinus patula* biochar in eliminating turbidity and COD from POME. The experiments were performed using a jar test to maintain a constant mixing and ensure effective interaction between the SS and OM compounds in POME and the biochar. Each beaker-type glass container in the jar test held 200 mL of POME with a known concentration of *Pinus patula* biochar.

Different biochar doses (2, 4, and 6 g/L) and pH values (3, 6, and 9) were tested to determine the optimal conditions for the removal of turbidity, intended as the response variable during optimization procedure, which was maximized. Based on the PZC of the *Pinus patula* biochar (PZC = 6) [44], the values of pH were selected.

After adding *Pinus patula* biochar to the solution, the pH was adjusted using 1 M NaOH and 1 M HCl. A contact time of 60 min was established for the experimental design matrix, and 20 mL aliquots were withdrawn at 30 and 60 min of contact time using syringes. Since turbidity removal efficiencies were comparable between both contact times across the treatments evaluated, a contact time of 30 min was selected for optimization purposes, favouring process efficiency without compromising treatment performance and avoiding excessive hydraulic retention times. The samples were then filtered with a vacuum pump and cellulose filters with a pore size < 375 μm to assess turbidity removal. The change in turbidity was analyzed using a Turbiquant™ 1100 T Turbidimeter (Merck KGaA, Germany), in which turbidity values were registered based on the intensity of the scattered light [31].

Turbidity removal efficiency, *E* (%), was determined as described in Equation (2), where *T*_0_ is the initial turbidity of the POME sample, and *T_f_* is the turbidity of the POME after the treatment with *Pinus patula* biochar for each treatment time.

(2) $$E\left(\%\right)=\frac{T_{0}-T_{f}}{T_{0}}\times 100$$

#### Design of Experiments

A face-centred CCD was used to analyze the effect of the operational parameter (i.e., solution pH and biochar dose). For each operational factor, three levels were evaluated. As presented in Table 8, the levels for each operating parameter were coded as 0 (the centred point), +1 (the highest level), and −1 (the lowest level). The optimization of the treatment of POME using *Pinus patula* biochar was assessed using turbidity removal as the response variable, which was maximized.

To fit the experimental data and evaluate the primary, interaction, and quadratic effects of each operating parameter, a quadratic regression model was selected [66]. The model is expressed in Equation (3), where *y* is the predicted turbidity elimination efficiency, *β*_0_ is the constant value, *β*_1_ and *β*_2_ are the linear coefficients of the primary effects, related to the pH of the solution and biochar dose, respectively; *β*_12_ is the coefficient of the effect of the interaction between both factors, and *β*_11_ and *β*_22_ are the coefficients of the square effect of each operational parameter. Considering that the resulting design follows a 3^2^ CCD, a total of 11 experiments were conducted, including the central points. To minimize bias and ensure independency of observations, the treatments were performed in a randomized order [34].

(3) $$y=\beta_{0}+\beta_{2}x_{2}{+\beta }_{1}x_{1}+\beta_{12}x_{1}x_{2}+\beta_{11}{x_{1}}^{2}+\beta_{22}{x_{2}}^{2}$$

The software Statgraphics Centurion version XVIII (StatPoint Technologies, Warrenton, VA, USA) was used to determine the optimal pH and *Pinus patula* biochar dose for maximizing turbidity removal from POME. To evaluate the significance of the operational factors and the regression model, an ANOVA was conducted, and a *p*-value < 0.05 (α = 5%) was considered. The model fit was assessed using the correlation coefficient (*R*^2^) and its adjusted value (*R*^2^*_adj_*) based on the degrees of freedom [34]. Additionally, the assumptions of homoscedasticity, normality, and residual independence were tested using the software R version 4.5.2 (R Core Team, Iowa city, IA, USA) to ensure the validity of the quadratic model built.

## 4. Conclusions

This study demonstrates that biochar from *Pinus patula* can be an alternative for treating POME, achieving turbidity and COD elimination rates of 58.27% and >50%, respectively, under optimal conditions (6 g/L biochar dose and solution pH of 6) and a retention time of 30 min. Furthermore, this method promotes circular bioeconomy by repurposing solid waste, thereby contributing to more efficient and responsible waste management.

Solution pH was identified as the most significant parameter affecting turbidity removal, exhibiting a non-linear/quadratic effect with a pronounced impact at intermediate values. At a pH of 6, biochar operates at a neutral charge, favouring interactions with contaminants without relying on electrostatic forces. Maintaining this pH value allows for the optimization of contaminant removal along with the elimination of the need for additional pH adjustments and associated costs, as it falls within the maximum permissible range established by Colombian discharge regulations. Regarding biochar concentration, a dose of 6 g/L proved to be the most effective; at higher doses, the increased active site availability and surface area enhance the removal of turbidity-causing substances.

The findings of this study confirm that biochar produced from *Pinus patula* is effective in pollutant removal from POME and contributes to sustainable resource management. However, future research should focus on optimizing the treatment of POME with biochar at an industrial scale, evaluating its performance under real operating conditions, and comparing it with other adsorbents. Additionally, it is essential to study the ability of biochar to remove a broader range of pollutants and to perform life cycle analyses to assess its overall sustainability. Prioritizing the use of waste, such as forestry residues, as a raw material to produce biochar is recommended to further foster circular bioeconomy and adjust solutions to local conditions.

## Figures and Tables

**Table 1:** Initial physico-chemical characterization of palm oil mill effluent (POME).

Parameter	Unit	Value
Turbidity	NTU	296 ± 8
pH	-	7.93 ± 0.5
Conductivity	mS/cm	0.21 ± 0.05
Temperature	°C	24.3 ± 0.5
Chemical oxygen demand (COD)	mgO_2_/L	15,400 ± 1758.63

**Table 2:** Results of *Pinus patula* wood pellets and biochar characterization.

Property	Units	*Pinus patula* Wood Pellets	*Pinus patula* Biochar
Moisture content	%wt	7.91	11.13
Fixed carbon		14.09	77.49
Volatile matter		84.64	20.59
Ash content		1.27	1.92
C		47.01	97.94
O		47.28	0.9
H		5.69	0.97
N		0.02	0.19
Pore volume	cm^3^/g	0.0006	0.20
Specific surface area	m^2^/g	1.16	367.33

**Table 3:** Face-centred central composite design CCD matrix predicted and experimental values for turbidity elimination by *Pinus patula* biochar. Operating conditions: pH = 9, 6, and 3; biochar dose = 2, 4, and 6 g/L; contact time = 30 min; biochar particle size = 375 µm.

Treatment	Operational Parameter		Turbidity Removal (%)	
	pH (-)	Biochar Dose (g/L)	Predicted Value	Experimental Value
1	3	2	39.31	39.45
2	9	2	24.06	22.88
3	3	6	42.13	43.55
4	9	6	32.88	32.99
5	3	4	41.35	39.79
6	9	4	29.10	30.17
7	6	2	50.56	51.59
8	6	6	56.38	54.85
9	6	4	54.10	60.59
10	6	4	54.10	55.30
11	6	4	54.10	46.92

**Table 4:** ANOVA for the elimination of turbidity from POME by *Pinus patula* biochar. Operating conditions: pH = 9, 6, and 3; biochar dose = 2, 4, and 6 g/L; contact time = 30 min; biochar particle size = 375 µm.

Source	SS ^1^	DF ^2^	MS ^3^	F ^4^	*p* ^5^
A: pH	225.09	1	225.09	12.67	0.0119
B: Biochar dose	50.86	1	50.866	2.86	0.1415
AA	989.21	1	989.21	55.70	0.0003
AB	9.03	1	9.03003	0.51	0.5026
Total error	106.56	6	17.759		
Total (corr.)	1380.76	10			

^1^ Sum of squares, ^2^ Degrees of freedom, ^3^ Mean square, ^4^ F-ratio, and ^5^ *p*-value.

**Table 5:** Comparison of removal mechanisms and efficiencies of several adsorbents used in POME treatment.

Adsorbent	Initial Conditions	pH Condition	Adsorption Mechanisms	Removal Efficiency	Ref.
Biochar montmorillonite-TiO_2_ composite	COD = 1256 mgO_2_/L Turbidity = 738 FAU	Near-neutral pH	Reduced influence of electrostatic forces, physical adsorption	COD = 56%	[45]
Oil palm truck derived biochar	COD = 2850 mgO_2_/L	pH = 8	Pore filling	COD = 4.38%	[30]
Raw crab waste shell biochar activated with microwave steam	--	pH = 10	Pore filling, physical adsorption	COD = 49%, 42 mg/g	[46]
Ground biochar/TiO_2_/Polylactic acid composite	COD = 2584 mgO_2_/L Turbidity = 1876 NTU	pH = 4.4	Pore filling, physical adsorption	Turbidity = 61.5% COD = 39%	[47]
Granular-activated carbon	pH = 4.25 COD = 55,550 mgO_2_/L	pH = 4	Electrostatic attraction	COD = 88%	[48]
Oil palm kernel shell bioadsorbent	COD = 604 mgO_2_/L	pH = 8	Pore filling	COD = 68%	[49]
*Pinus patula* biochar	COD = 15,400 mgO_2_/L Turbidity = 268 NTU	pH = 6	Pore filling, physical adsorption	Turbidity = 58.27% COD ≥ 50%, 1309 mg/g	This study

**Table 6:** Statistics for the validation of the quadratic regression model built.

Model Validation Statistics	Value
R^2^	0.93
R^2^_adj_	0.87
*p*-value	0.0017
Durbin–Watson test	0.61
Breusch–Pagan test	0.45
Shapiro–Wilk test	0.11

**Table 7:** Reported studies on the elimination of turbidity from POME.

Treatment	Operational Conditions	Turbidity Removal Efficiency (%)	Ref.
Electrocoagulation	t = 12 h Voltage = 6.5 V pH = 7.0 T = 27 °C	99 (synthetic water)	[1]
Electrochemical peroxi-coagulation	t = 20 h H_2_O_2_ dose = 31.09 mM Voltage = 1.5–2.5 V pH = 3	84 (synthetic water)	[2]
Flocculation–coagulation with pomegranate seeds	t = 25 min Coagulant dose = 2391.91 mg/L pH = 10.37	98 (synthetic water)	[58]
Ultrafiltration	t = 270 min Membrane = UF of 20 kDa P = 0.8 MPa pH = 4.52	88.5 (synthetic water)	[60]
Adsorption with *Pinus patula* biochar	t = 30 min Biochar dose = 6 g/L pH = 6 T = 25 °C	58.27 (real water)	This study

**Table 8:** Levels and operational parameters of the face-centred CCD.

Operational Factor	Levels		
	+1	0	−1
pH (−)	9	6	3
Biochar dose (g/L)	6	4	2

## Data Availability

The authors confirm that the data supporting the findings of this study are available within the article.
